# Esterase and Peroxidase Are Involved in the Transformation of Chitosan Films by the Fungus *Fusarium oxysporum* Schltdl. IBPPM 543

**DOI:** 10.3390/jof11080565

**Published:** 2025-07-29

**Authors:** Natalia N. Pozdnyakova, Tatiana S. Babicheva, Daria S. Chernova, Irina Yu. Sungurtseva, Andrey M. Zakharevich, Sergei L. Shmakov, Anna B. Shipovskaya

**Affiliations:** 1 Institute of Chemistry, Saratov National Research State University named after N.G. Chernyshevsky, 83 Astrakhanskaya St., Saratov 410012, Russia; pozdnyakova_n@ibppm.ru (N.N.P.); tatyana.babicheva.1993@mail.ru (T.S.B.); darianna1703@yandex.ru (D.S.C.); airinmind@yandex.ru (I.Y.S.); lab-15@mail.ru (A.M.Z.); shipovskayaab@yandex.ru (A.B.S.); 2 Institute of Biochemistry and Physiology of Plants and Microorganisms, Saratov Scientific Centre of the Russian Academy of Sciences (IBPPM RAS), 13 Entuziastov Prosp., Saratov 410049, Russia

**Keywords:** chitosan, transformation, *Fusarium oxysporum*, hydrolase, peroxidase

## Abstract

The majority of studies of fungal utilization of chitosan are associated with the production of a specific enzyme, chitosanase, which catalyzes the hydrolytic cleavage of the macrochain. In our opinion, the development of approaches to obtaining materials with new functional properties based on non-destructive chitosan transformation by living organisms and their enzyme systems is promising. This study was conducted using a wide range of classical and modern methods of microbiology, biochemistry, and physical chemistry. The ability of the ascomycete *Fusarium oxysporum* Schltdl. to modify films of chitosan with average-viscosity molecular weights of 200, 450, and 530 kDa was discovered. *F. oxysporum* was shown to use chitosan as the sole source of carbon/energy and actively overgrew films without deformations and signs of integrity loss. Scanning electron microscopy (SEM) recorded an increase in the porosity of film substrates. An analysis of the FTIR spectra revealed the occurrence of oxidation processes and crosslinking of macrochains without breaking *β*-(1,4)-glycosidic bonds. After *F. oxysporum* growth, the resistance of the films to mechanical dispersion and the degree of ordering of the polymer structure increased, while their solubility in the acetate buffer with pH 4.4 and sorption capacity for Fe^2+^ and Cu^2+^ decreased. Elemental analysis revealed a decrease in the nitrogen content in chitosan, which may indicate its inclusion into the fungal metabolism. The film transformation was accompanied by the production of extracellular hydrolase (different from chitosanase) and peroxidase, as well as biosurfactants. The results obtained indicate a specific mechanism of aminopolysaccharide transformation by *F. oxysporum*. Although the biochemical mechanisms of action remain to be analyzed in detail, the results obtained create new ways of using fungi and show the potential for the use of *Fusarium* and/or its extracellular enzymes for the formation of chitosan-containing materials with the required range of functional properties and qualities for biotechnological applications.

## 1. Introduction

Chitosan is a biogenic amorphous–crystalline polymer of *N*-acetylglucosamine and glucosamine linked by *β*-(1,4)-glycosidic bonds with a wide range of biological activities [1,2]. Among its most discussed biological properties are antibacterial, antifungal, and immunostimulating activities [3,4,5]. Chitosan, as an elicitor, increases plant resistance to fungal and viral diseases, and also plays a key role in controlling plant immunity [6,7,8]. This aminopolysaccharide has fiber- and film-forming properties, which makes it a promising polymer for biotechnological applications, including carriers of pharmaceuticals and biocatalysts, biomedical engineering structures, microencapsulating agents for beneficial microorganisms, sorbents for cleaning industrial wastewater from metal compounds, new materials for agriculture, etc. [3,9,10]. One of the biotechnology areas is the production of chitosan-based biocomposites with novel properties after treating the original polymer with biological agents such as microorganisms and/or enzymes. The main approach to creating such biomaterials includes the chemical or physical immobilization of bio-objects in a polymeric matrix and its further transformation to form the required morphological form of the biomaterial (films, fibers, microcapsules, or hydrogels) [11,12]. Another approach is based on the enzymatic cleavage of *β*-(1, 4)-glycosidic bonds using, most often, glycosidases (chitosanase, hyaluronidase, cellobiohydrolase, etc.) for chemical modification of the polymer to form bioactive chitooligosaccharides [13,14,15]. Meanwhile, the most important functional quality in both the production and use of chitosan-containing biocomposites is their resistance to external influences with accessibility for structural, physical, or chemical transformation by microorganisms or enzymes in vivo, which cannot be implemented with the above-discussed approaches to polysaccharide modification [16]. In this regard, the development of new methods for the structural transformation of high-molecular-weight chitosan, including its physicochemical modification without breaking glycosidic bonds and maintaining the polymer substrate integrity, is relevant.

Fungi with the ability to actively degrade and transform a wide range of natural and synthetic polymers due to their complex of extracellular hydrolases and oxidoreductases are promising objects for the development of methods for obtaining such biomaterials/biocomposites [17,18]. Molecular technologies have shown that the main fungal players in the degradation and damage of various types of polymers are representatives of the Ascomycota phylum [19,20]. Representatives of the genus *Fusarium* are some of the most common ascomycetes, which are ubiquitous in soils and the rhizosphere of plants [21,22]. The majority of *Fusarium* species and strains are soil saprotrophs. They can utilize lignin, complex carbohydrates, plant residues, and even synthetic polymers as carbon sources, while producing a wide range of extracellular enzymes, including glycoside hydrolases, cutinases, oxidases (including laccases), etc. [21,23]. Chitosanases, the enzymes catalyzing the most rapid and profound hydrolysis of chitosan, have also been found in representatives of the genus *Fusarium* [24,25]. For example, the chitosanase from *F. solani* is an endo-chitosanase cleaving the polymer macromolecule at random sites to form chitooligosaccharides with a polymerization degree below ten [24]. The extracellular chitosanase from *F. oxysporum* was isolated and characterized for its main catalytic properties. The anticancer activity of chitooligosaccharides formed as a result of chitosan hydrolysis catalyzed by this enzyme has been shown [25]. Therefore, the production of a wide range of extracellular enzymes makes *Fusarium* sp. a promising object for the development of methods for obtaining new biotechnologically significant materials based on natural polymers.

The used strain *F. oxysporum* Schltdl. IBPPM 543 was isolated from old creosote-contaminated sleepers and exhibited high degradation activity towards a wide range of pollutants, including synthetic polymers such as polyethylene terephthalate [26,27]. We have proposed that this fungus, which produces a whole complex of extracellular oxidoreductases and hydrolases (other than chitosanases), could promote physicochemical modification of the polymer structure without losing the integrity and stability of the polymer material, which predetermines the possibility of both creating new functional products for various practical purposes and solving general problems of the rational use of natural resources.

In particular, we have hypothesized that *Fusarium* strains are capable of modifying the structure of chitosan to form chitosan-containing materials with a new set of functional properties and qualities for biotechnological applications. No changes have been studied in the structure, supramolecular ordering, and mechanical and physicochemical properties of any chitosan-containing biomaterial under conditions of its non-degradable fungal modification. In this regard, the aim of the presented work was to identify structural and physicochemical transformations of chitosan films after the growth of *F. oxysporum* Schltdl. IBPPM 543.

## 2. Materials and Methods

### 2.1. Substances and Reagents

In this work, three powdered chitosan (CS) samples of few average-viscosity molecular weights and close degrees of deacetylation were used (ZAO Bioprogress, Shchyolkovo, Russia, Table 1), along with 70% glycolic acid (Sigma-Aldrich, Saint Louis, MO, USA), sodium hydroxide (Khimreaktiv, Nizhny Novgorod, Russia), and distilled water. All reagents were used without further purification.

The ${\bar{M}}_{\eta }$ of chitosan was estimated by viscometry and calculated using the Mark–Kuhn–Houwink equation: $\left[\eta \right]\;=\;1.38\;\cdot \;10^{-4}\;\cdot \;{{\overset{-}{M}}_{\eta }}^{0.85}$ [28], where [η] is the intrinsic viscosity (dL/g) of a chitosan solution in Na-acetate buffer (0.33 M CH_3_COOH + 0.2 M CH_3_COONa, pH = 4.4) at 25.0 ± 0.1 °C. The degree of deacetylation was evaluated by standard potentiometric titration of chitosan hydrochloride with a NaOH solution, bulk density and moisture content were estimated gravimetrically, and ash content was obtained from the manufacturer’s data. The choice of samples with ${\bar{M}}_{\eta }\;=$ 200–530 kDa was due to chitosan exhibiting no fungicidal activity in this range of molecular weight [29].

### 2.2. Objects of Study

The objects of our study were chitosan films in the form of a polybase, before (initial, control films, CS-${\bar{M}}_{\eta }$) and after growth of the ascomycete *F. oxysporum* (experimental films, CS-${\bar{M}}_{\eta }$·*FO*). *F. oxysporum* (IBPPM 543) was obtained from the IBPPM RAS Collection of Rhizosphere Microorganisms (http://collection.ibppm.ru, accessed on 23 July 2022).

### 2.3. Preparing Chitosan Films

To obtain films, chitosan solutions with concentrations of 3.5–4 wt.% in 1.5% glycolic acid were prepared under stirring on a magnetic stirrer at 22 ± 2 °C for 6–7 h, followed by keeping at room temperature for 18–19 h to remove air bubbles. Our choice of glycolic acid rather than traditional chitosan solvents (CH_3_COOH, HCOOH, and HCl) was due to its pharmacological activity, which allows obtaining materials with improved biochemical and physicomechanical properties [30,31]. Films were obtained by pouring the chitosan solution onto a polished polyethylene terephthalate substrate (Petri dish), followed by drying for 6–7 days in an air atmosphere at 22 ± 2 °C. To obtain films of uniform thickness, the volume of the solution and the concentration of chitosan were varied depending on its ${\bar{M}}_{\eta }$. To convert chitosan glycolate into the basic form, the film samples were kept in a 5% NaOH solution for 3 h, washed with distilled water for 6–7 days until the pH of the wash water was 7, and dried in room atmosphere until air-dried samples were obtained.

### 2.4. F. oxysporum Cultivation on Chitosan Films

The fungus was grown on a modified basidiomycetes-rich medium (g/L): NH_4_NO_3_, 0.724; KH_2_PO_4_, 1.0; MgSO_4_·7H_2_O, 1.0; KCl, 0.5; yeast extract, 0.5; FeSO_4_·7H_2_O, 0.01; ZnSO_4_·7H_2_O, 0.0028; CaCl_2_·2H_2_O, 0.033; glucose, 10.0; peptone, 10.0; and agar, 15.0 (pH 6.0) [32]. To grow the fungus in the presence of chitosan films, whose aminopolysaccharide base was the sole source of carbon and energy, only the salt base of the same medium was used (glucose, peptone, and yeast extract were excluded from the medium). The films were crushed into 0.5×0.5 cm fragments, sterilized with ethanol for 60 min, and the remaining alcohol was removed with sterile filter paper. A total of 250 mg of the film sample and 5 mL of the fungal inoculum were added to flasks with 100 mL of the medium. They were cultured at 25 and 30 °C for 30 days and stirred at 130 rpm in a New Brunswick™ Excella^®^ E24/E24R Shaker incubator (Eppendorf, Nürtingen, Germany). Optical density (*A*) was measured spectrophotometrically at 600 nm using an Evolution 60 spectrophotometer (ThermoScientific, Waltham, MA, USA). At certain time intervals, an aliquot (2 mL) was taken from the flasks to evaluate enzyme activity. The fungal mycelium growth in the culture liquid was measured gravimetrically at the end of the experiment [33].

### 2.5. Examination of the Physicochemical Characteristics of Chitosan Films

After the micromycete *F. oxysporum* growth was completed, the chitosan films were soaked in distilled water for 2 days and dried in a UT 46-83 oven (Ulab, Nanjing, China) to an air-dried state.

Thickness (μm) was measured with a digital micrometer RGK MC-25 (RGK, Russian Federation), with a division value of 0.001 mm, expressed as the arithmetic mean of 10 measurements at several areas of the film sample. Moisture content (%) was determined on an MX-50 moisture analyzer (AND, Tokyo, Japan), with an accuracy of 0.1%, expressed as the arithmetic mean of 5 measurements. Color was assessed visually according to the RAL color standard.

If necessary, the preparations were dispersed on a Fritsch PULVERISETTE 7 Premium Line planetary micromill (FRITSCH, Idar-Oberstein, Germany) in a 20 mL grinding jar using tungsten carbide grinding balls (diameter 5 mm, 40 pcs.) at 300 rpm for 1 h.

Solubility was estimated gravimetrically. Dispersed samples were kept in a Na-acetate buffer with pH 4.4 (at a molar ratio of the buffer and amino groups –NH_2_ in the chitosan sample of ≥10) at 22 ± 2 °C for 1 day with periodic stirring on a magnetic stirrer. The insoluble part was quantitatively separated by filtration through an ash-free filter “Blue Ribbon” (MELIOR XXI Ltd., Moscow, Russia). The filtrate was centrifuged in a CM-70M-07 centrifuge (LLC ELMI, Riga, Latvia) at 9000 rpm for 1.5 min, and the supernatant was removed. The sediments were combined and dried to constant weight in a UT 46-83 oven (Ulab, China). The mass of the insoluble fraction (CS-${\bar{M}}_{\eta }$·*FO^InsF^*, %) was calculated taking into account the moisture content in the original preparation.

### 2.6. Methods for Testing Chitosan Films

Gravimetric measurements were carried out on Ohaus Adventurer AR 1530 scales (accuracy: ±0.00002 g, Ohaus, Mumbai, India).

#### 2.6.1. Microscopy

Electron microscopic images were obtained by scanning electron microscopy (SEM) on a MIRA\\LMU microscope (Tescan, Brno, Czech Republic) at a voltage of 15 kV and a conducting current of 400 pA. A 5 nm thick gold layer was deposited onto each sample using a K450X Carbon Coater (Emitech, Chelmsford, England).

#### 2.6.2. Physicomechanical Tests

Physicomechanical properties were tested using a universal tensile testing machine Tinius Olsen H1KT-S (Tinius Olsen, Redhill, UK). Breaking stress (σ_p_, MPa), relative elongation at break (ε_p_, %), and Young’s modulus (*E*, MPa) were calculated using standard methods taking into account the cross-sectional area, initial length, and thickness of the original preparation, expressed as the arithmetic mean of 6 measurements.

#### 2.6.3. X-Ray Diffractometry

X-ray diffraction patterns were obtained on a DRON-8T diffractometer (JSC IC “Burevestnik”, Nizhny Novgorod, Russia) with CuK_α_ radiation, a Goebel parabolic mirror (AXO Dresden GmbH, Dresden, Germany), and a Mythen 2R1D position-sensitive detector with 640 channels (Dectris, Baden, Switzerland), with a resolution of 2θ = 0.0144 deg, in a 2 mm quartz cuvette. Focal beam geometry: an axial slit of 12 mm, and an equatorial slit of 0.25 mm. Registration was carried out in the angular range of 2θ = 5–40 deg by points, with a step of 0.02 deg for the central channel of the detector and an exposure time of 10 s per point. The degree of crystallinity (χ, %) was calculated as the ratio of the integrated intensity of the total scattering of crystallites to the total scattering from amorphous and crystalline regions by graphical integration (QCAD 3.15) [34].

#### 2.6.4. Fourier IR Spectroscopy

FTIR spectra of dispersed film samples were recorded on a Vertex 70v vacuum IR Fourier spectrometer (Bruker, Billerica, MA, USA), with PIKE GladiATR thermal variation and a resolution of 4 cm^−1^, using an average of 36 scans in the range of 4000–400 cm^−1^ by the ATR method. The spectra were processed by the OPUS 9.0 software. Vibrational absorption bands were identified according to standard correlation tables [35].

#### 2.6.5. Elemental Analysis

Elemental CHN analysis of dispersed film samples was performed on a Vario Micro Cube analyzer (Elementar, Germany): C, H—in a flow of O_2_, N—in a flow of CO_2_. The error was ±0.5 wt.%. C/N/H contents were calculated for an ash-free sample, taking into account the moisture content in the original or experimental chitosan powder.

#### 2.6.6. Atomic Absorption Spectrometry

The contents of Mg(II), Fe(II), and Zn(II) were determined on a Thermo Scientific iCE3500 atomic absorption spectrometer (ThermoScientific, USA). The films were dried at 70 °C to a constant weight and crushed, and 200 mg of each crushed film was taken for analysis by the quartering method. Preliminary acidic digestion of the samples was carried out in Teflon vessels in the presence of concentrated HNO_3_ and 30% H_2_O_2_ solution for 15 min. Mineralization was carried out under constant-temperature and pressure control on a closed-type MARSXpress microwave system (CEM, Matthews, NC, USA). The obtained samples were cooled, filtered, and analyzed.

#### 2.6.7. Sorption Capacity

Cu(II) sorption was carried out under static conditions in the thermostatting mode (25 ± 0.1 °C) using aqueous solutions of CuSO_4_·5H_2_O (reagent grade) and was monitored spectrophotometrically. Optical density was measured on a B-1100 VEK 2109010 spectrophotometer (Shanghai Mapada Instruments Co. Ltd., Shanghai, China) within a wavelength range of 350–700 nm in quartz cuvettes with an optical path length of 3 cm. The residual concentration of copper ions in the solution was determined using a pre-built calibration line. The sorption capacity (mg/g) was calculated based on the difference in ion concentration in the initial CuSO_4_ solution and after establishing sorption equilibrium, considering the mass of the film sample and the volume of the sorption medium.

### 2.7. Methods for Studying the Culture Liquid During F. oxysporum Growth on Chitosan Films

The emulsifying activity of the culture liquid was tested using the Cooper method [36]. The culture liquid was mixed with kerosene in a 2:3 ratio, shaken for 20 min in a measuring tube, and left at room temperature for 48 h for separation. Emulsifying activity (*E*_48_) was calculated as the ratio of the emulsion volume to the total volume of liquid and expressed as a percentage.

Enzyme activity was estimated using a standard method on an Evolution 60 spectrophotometer (Thermo Scientific, USA) in quartz cuvettes with an optical path length of 1 cm. Peroxidase or laccase activity was determined by the rate of formation of the oxidation product 2,2′-azino-*bis*(3-ethylbenzothiazoline-6-sulfate with (peroxidase) or without (laccase) 0.2 mM H_2_O_2_ at 436 nm [37]; esterase activity was estimated by *p*-nitrophenol formation during *p*-nitrophenylbutyrate hydrolysis at 410 nm [38]; chitosanase activity was studied according to Abedin et al. [25]. The amount of enzyme catalyzing the formation of 1 μmol of the reaction product per minute was taken as the unit of activity and expressed in arbitrary units—U/mL of the enzyme preparation.

The enzymatic activity for native films was detected on the example of a CS-200 film using a crude enzyme preparation from *F. oxysporum* containing peroxidase (2.3 U/mL) and hydrolase (1.49 U/mL); the protein concentration was 0.55 mg/mL. A total of 200 mg of a CS-200 film was placed into 7.5 mL of the crude enzyme preparation and incubated at 25 °C under 130 rpm for 4 days. The film after incubation with the crude enzyme preparation was designated as CS-200·*FO^En^*.

### 2.8. Statistical Analysis

Statistical analysis was performed for three to ten replicates of each experiment. The results are presented as mean values with standard deviations. The significant difference was evaluated by unpaired two-sample Student’s *t*-test. Any difference was considered statistically significant when *p* < 0.05.

## 3. Results

### 3.1. Use of Chitosan Films for Cultivating F. oxysporum as Its Sole Source of Carbon and Energy

#### 3.1.1. *F. oxysporum* Growth

**Figure 1:**
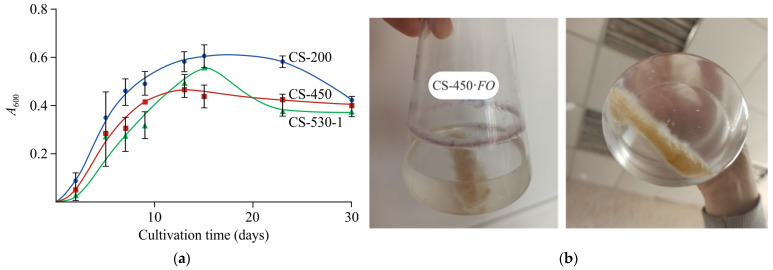
Growth curves of *F. oxysporum* in our culture medium at 25 °C, where CS-200, CS-450, and CS-530-1 films were the only carbon source; the confidence interval of the *A*_600_ values did not exceed 10% of the base value and is not shown in the figure (**a**); photos of *F. oxysporum* mycelium and the CS-450 *FO* film (**b**).

Chitosan films of few average-viscosity molecular weights (200, 450, and 530 kDa) and thicknesses (100 and 200 μm) were used as the sole source of carbon and energy while cultivation (Table 2 a). Active *F. oxysporum* growth was observed during the micromycete cultivation in a mineral medium in the presence of chitosan films (Figure 1a). In this case, no degradation of the films was observed, and biomass was accumulated both on the surface of the film substrates and as a colored ring of mycelium along the perimeter of the flask (Figure 1b). In all cases, regardless of ${\bar{M}}_{\eta }$, the fungus was in the logarithmic growth phase for approximately 10 days of cultivation, followed by the stationary phase. The decrease in absorption at *A*_600_ during cultivation may indicate active attachment of the mycelium to the film substrate surface or lysis of fungal cells. In the control treatment (a mineral medium without chitosan inoculated with the fungus), no growth of the micromycete was detected.

Our assessment of *F. oxysporum* biomass accumulation showed that, after 30 days of cultivation, the dry mycelium weight in different variants differed insignificantly and was 34.2 ± 13.3 mg, 30.0 ± 6.4 mg, and 30.8 ± 14.4 mg for CS-200, CS-450, and CS-530-1 films, respectively. Using the CS-530 polymer sample as an example, the effect of film thickness and cultivation temperature on mycelium formation was studied. It was found that a 2-fold decrease in film thickness (CS-530-1 and CS-530-2, Table 2 a) resulted in an insignificant (no more than 6 and 2%) increase in the amount of biomass in the cultivation medium at 25 and 30 °C, respectively. In the case of films of the same thickness, an increase in the cultivation temperature also resulted in an insignificant increase in mycelium production, namely, no more than 8 and 4% when the fungus grew on films with thicknesses of 100 and 200 μm, respectively. Since the fungal mycelium actively grew into the bulk of the films, it was not possible to reliably assess its total amount. The data presented relate only to the mycelium detected in the cultivation medium.

#### 3.1.2. SEM of the Film Surface

**Figure 2:**
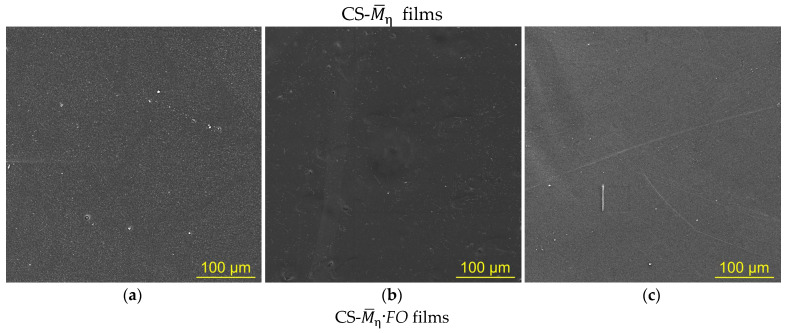

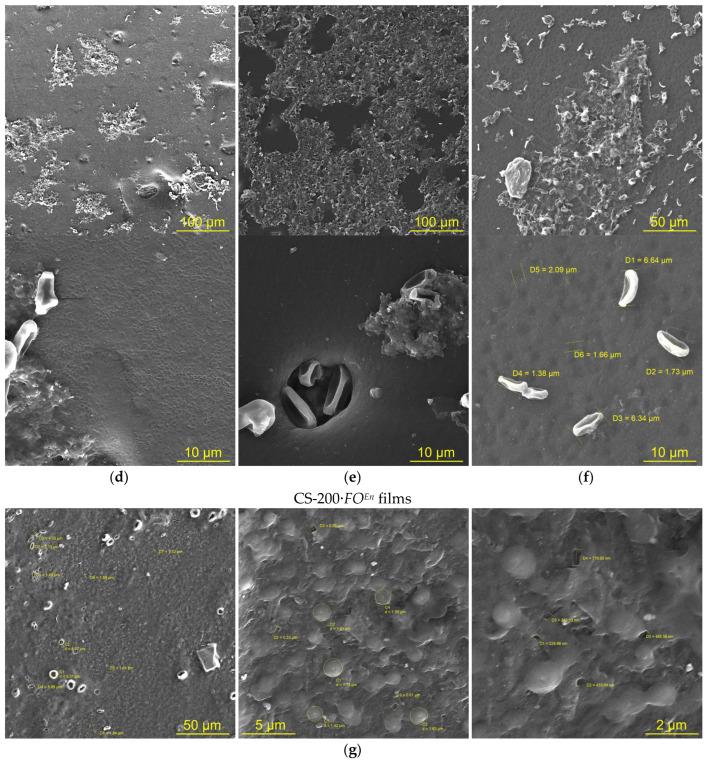
The SEM images of the surface of CS-200 (**a**,**d**,**g**), CS-450 (**b**,**e**), and CS-530-1 (**c**,**f**) films: initial ones ((**a**–**c**), CS-${\bar{M}}_{\eta }$), after *F. oxysporum* growth at 25 °C for 30 days ((**d**–**f**), CS-${\bar{M}}_{\eta }$·*FO*) and after incubation with the crude enzyme preparation from *F. oxysporum* at 25 °C for 4 days ((**g**), CS-200·*FO^En^*).

The SEM images of the chitosan films after *F. oxysporum* cultivation, as well as our visual observations (Figure 1b), revealed attachment of the fungus to the substrate surface (Figure 2a–f). The surface structure of the film samples was also transformed., e.g., the initial films were characterized by a homogeneous surface with slight roughness (Figure 2a–c). After *F. oxysporum* growth, defects such as hemispherical pores were observed on the films (Figure 2d–f). Pores uniformly covered the entire surface of the film samples. The average diameter of such pore-like defects varied in a range of ~1–3 μm. Large pores with diameters within ~5–15 μm were also detected, inside which particles of the fungal mycelium were clearly visualized. The most developed surface morphology with the highest total number of defects was observed for the CS-200·*FO* sample.

### 3.2. Chitosan Film Properties After F. oxysporum Growth

#### 3.2.1. Physicochemical Properties

At the end of the experiment, after *F. oxysporum* growth, a decrease in the mass of air-dried films and a significant increase in their thickness compared to the control without the fungus were observed (Table 2a,b). The change in this geometric parameter was not associated with an increased moisture content, since the experimental films were dried to the moisture content of the original film samples (Table 2c). The observed increase in the thickness of CS-${\bar{M}}_{\eta }$·*FO* films may be due to both mycelial germination into the bulk of the film sample and structural transformation of the polymer substrate. In this regard, it was not possible to reliably measure the decrease in the dry mass of chitosan in our experimental films.

Visual inspection of the chitosan films after *F. oxysporum* growth showed the absence of destructive deformation changes such as cracks, chips, clouding, twisting, etc. The above-described pore-like defects were only visible at high magnification using SEM (Figure 2d–f). A change in the color of the films from light beige for CS-${\bar{M}}_{\eta }$ to ocher-brown for CS-${\bar{M}}_{\eta }$·*FO* was also noted (Table 2d). An unexpected result was the almost complete loss of solubility of the experimental samples in the Na-acetate buffer (pH 4.4), which is a classic dissolving medium for chitosan (Table 2e). Regardless of the temperature conditions of fungal cultivation, the insoluble fraction content in the CS-530·*FO* films was higher than that in the chitosan films with a lower molecular weight.

Another feature is that the CS-${\bar{M}}_{\eta }$·*FO* films were ground to a finely dispersed state neither by manual mechanical abrasion nor in an electric mill. Dispersion of the samples to microparticles was possible only as a result of an hour-long exposure to a ball micromill under conditions of high rotation speed of the planetary disk. At the same time, the initial chitosan films were relatively easily dispersed by standard grinding in a porcelain mortar.

Since the significant loss of solubility and the acquired resistance to abrasion exceeded the effect that might be associated only with some transformation of a thin surface layer of the film material, the results obtained indicate changes in the supramolecular and, possibly, chemical structure of the polymer in our experimental films. This predetermined our study of the physicomechanical properties and supramolecular and chemical structure of the CS-${\bar{M}}_{\eta }$·*FO* films.

#### 3.2.2. Physicomechanical Properties

**Figure 3:**
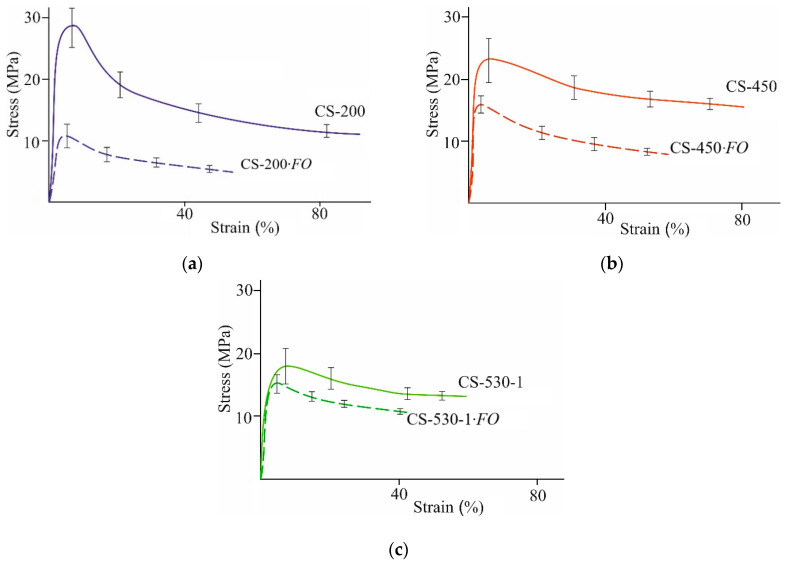
Stress–strain curves of CS-200 (**a**), CS-450 (**b**), and CS-530-1 (**c**) films: the initial ones (CS-${\bar{M}}_{\eta }$) and after *F. oxysporum* growth at 25 °C for 30 days (CS-${\bar{M}}_{\eta }$·*FO*).

Evaluation of the deformation–strength behavior, characterizing the properties and structural features of our films, showed the identity of the deformation pattern of all the studied CS-${\bar{M}}_{\eta }$ and CS-${\bar{M}}_{\eta }$·*FO* samples under uniaxial tension (Figure 3). The deformation–strength curves of the initial and experimental films were typical of amorphous–crystalline polymers in the glassy state. The film samples were stretched non-uniformly with neck formation due to orientation of macromolecular structures (the yield effect) without subsequent strain hardening. However, the CS-${\bar{M}}_{\eta }$·*FO* films, after *F. oxysporum* cultivation, were deformed more uniformly and, despite the above-mentioned resistance of the films to mechanical dispersion, exhibited significantly lower tensile strength than the original ones, not exposed to the fungus. For our experimental film samples with all ${\bar{M}}_{\eta }$’s, a decrease in tensile strength (σ_p_), relative elongation at break (ε_p_), and Young’s modulus (*E*) was observed (Table 2 g–i). This nature of the change in the deformation–strength characteristics was somewhat unexpected, since, according to classical laws, a decrease in the strength of polymer films is usually accompanied by an increase in their elasticity. The simultaneous decrease in both strength and elasticity of chitosan films may be associated with various chemical modifications of the polymer. For example, the films made of chitosan modified with disulfide bonds exhibited deformation behavior similar to the CS-${\bar{M}}_{\eta }$·*FO* films [2]. The modification with thioctic acid was carried out by reacting the amino groups of the polysaccharide with the carboxyl groups of the acid to form amide bonds. The authors also noted the loss of solubility of such films not only in standard water–acid organic media (chitosan solvents) but also under high acidity conditions (pH 1).

The strength characteristics of our film samples after *F. oxysporum* growth depended on the molecular weight of chitosan. E.g., the tensile strength had the lowest values for the experimental CS-200·*FO* films (Table 2g). For the same films, SEM recorded the highest total number of surface defects (Figure 2d). The lowest values of relative elongation at break and Young’s modulus were found in the experimental films of high-molecular-weight chitosan, namely, CS-450·*FO* and CS-530-1·*FO* (Table 2h,i). It seems natural that, for the initial CS films with this ${\bar{M}}_{\eta }$, the values of ε_p_ and *E* were also lower than for the initial CS-200 films. This indicates a greater structural heterogeneity of the control films CS-450 and CS-530 compared to CS-200, which is typical for thin films of high-molecular-weight chitosans [39].

Taken together, the decrease in the strength and elasticity of the CS-${\bar{M}}_{\eta }$·*FO* films under uniaxial tension compared to the CS-${\bar{M}}_{\eta }$ control may be due to an increase in the cross-sectional area of the samples (Table 2 a), as well as the appearance of surface and bulk porous microdefects due to transformation of the polymer morphostructure under the influence of *F. oxysporum* (Figure 2d–f).

#### 3.2.3. Supramolecular Ordering

**Figure 4:**
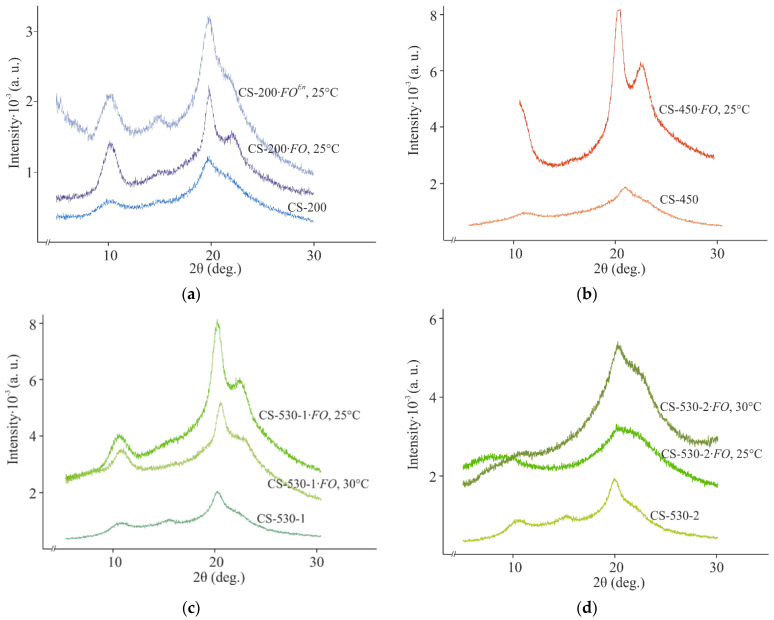
X-ray diffraction patterns of CS-200 (**a**), CS-450 (**b**), CS-530-1 (**c**), and CS-530-2 (**d**) films: the initial ones (CS-${\bar{M}}_{\eta }$), after *F. oxysporum* growth (CS-${\bar{M}}_{\eta }$·*FO* at 25 and 30 °C) for 30 days and after incubation with the crude enzyme preparation from *F. oxysporum* (CS-${\bar{M}}_{\eta }$·*FO^En^*) at 25 °C for 4 days.

Our study of supramolecular ordering showed that the initial chitosan films were characterized by an isotropic amorphous–crystalline structure with a low degree of crystallinity, χ = 30–40% (Figure 4, CS-${\bar{M}}_{\eta }$; Table 2f). The interplanar distances in the crystal lattices corresponded to those in the typical lattice of polymorphic modifications of the basic chitosan films obtained by treating freshly formed films with a NaOH solution, followed by washing with water and drying [34,40] with the main reflections at 2Θ~10.1–10.5 and 19.7–20.5 deg, as well as a weak reflection at 2Θ~15.0–15.7 deg (Table S1).

The structural ordering of our chitosan films after fungal cultivation differed significantly from that of the control samples. E.g., the X-ray diffraction patterns of the 200 μm thick CS-200·*FO*, CS-450·*FO,* and CS-530-1·*FO* films after *F. oxysporum* growth at 25 °C were characterized by an increase in the intensity and a slight broadening of the main reflections at 2Θ~9.9–10.3 and 19.7–19.8 deg, which indicated the presence of smaller structural elements therein (Figure 4a–c, CS-${\bar{M}}_{\eta }$·*FO*, 25 °C; Table S1). A diffuse reflection at 2Θ~14.9 deg was revealed for the CS-200·*FO* film only. In addition, a new reflection at 2Θ~21.8–22.1 deg appeared on the equatorial X-ray profiles of the samples. The occurrence of the latter indicates the formation of a polymorph with more straightened and closely spaced chains. According to Naito et al. [41], such modifications may occur during the crystalline transition of hydrated chitosan to its dehydrated form, for example, during hydrothermal treatment. The change in the orientation of macromolecular chains and the size of crystallites in the experimental CS-${\bar{M}}_{\eta }$·*FO* films was confirmed by a significant increase in the degree of crystallinity of the polymer, χ = 44–57% (Table 2 f). Consequently, under the micromycete influence, the amorphous phase of chitosan was transformed and the degree of ordering of the polymer structure increased. Using the example of CS-530-1, it was shown that a similar nature of changes in the supramolecular structure of the experimental films with a thickness of 200 μm was also observed under conditions of fungal cultivation at 30 °C (Figure 4c, CS-${\bar{M}}_{\eta }$·*FO*, 30 °C; Table 2 f; Table S1).

A somewhat different character of supramolecular transformation was observed for the films with an initial thickness of 100 μm (Figure 4d; Table 2 f; Table S1). After *F. oxysporum* cultivation at 25 °C, some amorphization of the chitosan structure in the film substrate was observed with a slight decrease in the degree of crystallinity of the polymer. However, when culturing at 30 °C, a structure formation variant almost similar to the CS-${\bar{M}}_{\eta }$·*FO* samples with a thickness of 200 μm was observed for CS-530-2·*FO*. E.g., an increase in the intensity of the main reflections was observed at 2Θ~10.3 and 20.2 deg. A blurred equatorial reflection was also revealed at 2Θ~22.4 deg, superimposed on the reflection at 2Θ~20.2 deg. The degree of crystallinity of the CS-530-2·*FO* film was somewhat higher than for the initial CS-530-2 sample, but the scale of the increase in χ was significantly smaller compared to the CS-${\bar{M}}_{\eta }$·*FO* films with the initial thickness of 200 μm. All this indicates that the ordering in the mutual arrangement of the CS-530-2·*FO* macromolecules is limited to a relatively small number of neighboring segments. Probably, the thickness of the 100 μm CS-530-2·*FO* film was insufficient for the stable formation of fungal mycelium on the film substrate surface at 25 °C and, accordingly, the structural transformation of chitosan compared to the same sample at 30 °C or the CS-${\bar{M}}_{\eta }$·*FO* films of a 200 μm thickness. It is possible that, in the presence of CS-530-2, *F. oxysporum* grows more actively at 25 °C in the bulk of the culture medium.

In combination, the physicochemical and physicomechanical properties, as well as the features of the supramolecular ordering of our experimental films, indicate the transformation of the films by *F. oxysporum* and some specific nature of the micromycete interaction with the polymer.

### 3.3. Identification Studies on the Chitosan Films After F. oxysporum Growth

#### 3.3.1. IR Spectroscopy

**Figure 5:**
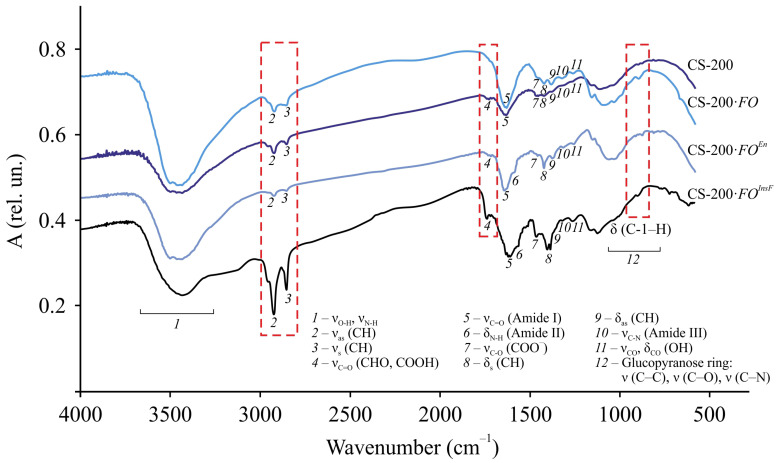
FTIR spectra of dispersed samples of the original CS-200 film, CS-200·*FO* film, and its insoluble fraction CS-200·*FO^InsF^* after *F. oxysporum* growth at 25 °C for 30 days and CS-200·*FO^En^* film after incubation with the crude enzyme preparation of *F. oxysporum* at 25 °C for 4 days. The correlation of the absorption bands is given in Table S2.

Typical FTIR spectra and the correlation of absorption bands of the original samples, the films after *F. oxysporum* cultivation, and their insoluble fraction are presented using CS-200 as an example (Figure 5, Table S2). The spectra of all the preparations contain few absorption bands typical for chitosan, namely, overlapping stretching vibrations of –OH and –NH groups bound by hydrogen bonds (3700–3300 cm^−1^, broad), stretching vibrations of –CH bonds of the methylene (2925–2922 cm^−1^, antisymmetric) and methyl groups (2857–2853 cm^−1^, symmetric), Amide I (1633–1630 cm^−1^, intense) and Amide III (1323–1313 cm^−1^, weak), deformation bond vibrations in the –CH_2_– and –CH_3_ fragments (1422–1400 cm^−1^, scissor; 1385–1381 cm^−1^, antisymmetric), planar stretching and deformation vibrations of the O–H bond (1262–1261 cm^−1^, weak), and stretching and deformation vibrations of the glucopyranose ring bonds (1170–840 cm^−1^). For all samples, a characteristic frequency corresponding to the deformation vibrations of C-1–H in *β*-sugars (896 cm^−1^, average) was detected in the range of glucopyranose ring bands. An absorption band of stretching vibrations of C–O in ionized carboxyl groups (1460 cm^−1^, average) was also detected, which, considering the ash content in the original chitosan (Table 1), indicates the presence of a small amount of metal ions in the samples.

A distinctive feature of the FTIR spectra of the CS-200 film after *F. oxysporum* growth and its insoluble fraction from that of the original sample is a shift of the characteristic frequencies of the stretching (ν_as_, ν_s_) and deformation vibrations (δ_as_, δ_sc_) of the C–H bond in the methylene and methyl groups, the stretching vibrations of C=O in the –NHCO– fragment (Amide I) with splitting of this band for the CS-200·*FO^InsF^* sample, and the stretching vibrations of C–N in the amino group (Amide III). For the insoluble residue, a significant increase in the intensity of the ν_as_, ν_s_, and ν_C=O_ bands was also observed, which, with regard to the C=O vibration band of the amide group, indicated crosslinking of chitosan chains. In addition, the spectrum of CS-200·*FO^InsF^* contained an Amide II band (1560 cm^−1^, bend), determined mainly by N–H bond stretching. Probably, during the structural transformation of our chitosan films under the micromycete influence, the conjugation of the N–H bond with the stretching vibrations of the C=O bonds was significantly reduced. In addition, in the spectral lines of the CS-200·*FO* and CS-200·*FO^InsF^* samples, in contrast to the original chitosan, an absorption band was found at 1736 cm^−1^, which was most clearly revealed for the insoluble fraction of the film, related to the stretching vibrations of the C=O of aldehyde and/or carboxyl groups. Note that such a band was revealed in the FTIR spectra of oxidized chitosans [42,43]. Therefore, the formation of these groups may be associated with oxidation reactions occurring primarily in the accessible amorphous phase of the film substrate. The discovered facts of chemical chitosan transformation are consistent with the change in the reflective properties of the experimental CS-200·*FO* samples, a decrease in transmission in the FTIR spectra (Figure 5), a color change (Table 2 d), and an increase in the degree of crystallinity (Table 2 f).

Similar changes in the FTIR spectra were recorded for the CS-450 and CS-530-1 films and their insoluble fraction after *F. oxysporum* cultivation (Figure S1).

Based on the FTIR spectroscopy data and taking into account that, for the insoluble fraction, the intensity and resolution of the absorption bands ν_as_(CH) and ν_s_(CH) are significantly higher (especially for the stretching vibration –CH_2_– of the pyranose ring), and δ_sc_(CH) is shifted by almost 22–37 cm^−1^ to the low-frequency region compared to the control sample, it can be assumed that the main centers of chemical transformations are the C-2 and C-6 atoms of the glucopyranose cycle. Possible participation of the group of atoms at C-3, for example, in oxidation processes cannot be ruled out. The latter, in turn, may lead to rupture of the bonds between C-2 and C-3 in the glucopyranose unit. Since the intensity and frequency of oscillations of the δ(C-1–H) band of the original and experimental samples are the same, rupture of the *β*–1, 4–glucosidic bonds of macrochains under the influence of *F. oxysporum* is unlikely. The preservation of the integrity of the film samples throughout the entire cultivation period serves as an indirect confirmation.

#### 3.3.2. Organo-Element Analysis (Elementary Glucopyranose Unit)

The CHN compositions of the CS-200·*FO* and CS-450·*FO* samples were analyzed. The powder obtained by dispersing the CS-530·*FO* film on a planetary micromill exhibited a high triboelectric effect, so it was not possible to obtain reliable information on the content of elements therein.

Quantitative C/N/H analysis showed a reliable decrease in the content of all elements in the experimental chitosan films compared to the control (Table 3). The decrease in the percentage of carbon and nitrogen in the samples after *F. oxysporum* growth indicates that not only carbon-containing fragments of the elementary units of the macrochain but also their nitrogen-containing groups were involved in chitosan transformation. In addition, the significantly higher C/N mass ratio in the CS-${\bar{M}}_{\eta }$·*FO* films than in the control ones indirectly indicates a partial loss of N-containing fragments. The slight decrease in the hydrogen percentage may be due to an oxidation reaction, probably due to the conversion of hydroxyl groups into carbonyl ones with a simultaneous hydrogen loss. At the same time, the decrease in the C/H mass ratio (due to the simultaneous decrease in the C and H content in our experimental samples) indicates some violation of the overall chemical structure of chitosan. The most significant changes in the chemical structure were recorded for the CS-200·*FO* film.

#### 3.3.3. Elemental–Inorganic Analysis (Metals)

**Figure 6:**
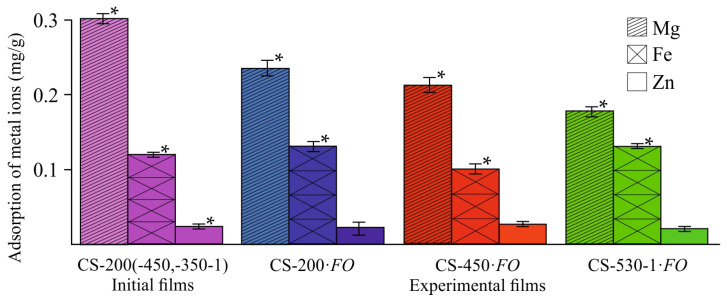
Changes in the content of metal ions in the CS-${\bar{M}}_{\eta }$·*FO* films after *F. oxysporum* growth at 25 °C for 30 days. The control corresponds to the average contents of Mg, Fe, and Zn in the film samples CS-200, CS-450, and CS-530-1. (*) means a statistically significant difference with the same indicator in the control experiment (*p* ≤ 0.05).

For the normal functioning of fungal cells, metal ions are necessary, which are either tightly bound in the composition of metalloenzymes (Zn in metalloenzymes without prosthetic groups; Fe in chelate complexes with porphyrin or heme in oxidases or peroxidases), or serve as activators of various enzymatic reactions (Mg during glycolysis). The cultivation medium of *F. oxysporum* contained sulfates of Mg (1 g/L), Fe (10 mg/L), and Zn (2.8 mg/L). These metals were also found in the structure of the original CS-${\bar{M}}_{\eta }$ films (Figure 6, CS-${\bar{M}}_{\eta }$). However, Mg, Fe, and Zn were present in small quantities, and their content was almost independent of the ${\bar{M}}_{\eta }$ of the polymer, which was consistent with the ash content of the initial chitosan powders (Table 1) and the FTIR spectra of the control and experimental films (Figure 5 and Figure S1, the ν_C–O_ band in carboxylate ions).

Despite the fact that chitosan exhibits a high sorption capacity for metal ions, including Fe^2+^ [44,45], our studies showed a slight increase in the Fe content in the experimental CS-200 and CS-530-1 films, not exceeding 1.0–1.2% of its mass fraction in the original CS powder, and a decrease in the CS-450·*FO* sample (Figure 6, CS-${\bar{M}}_{\eta }$·*FO*). As expected, the Zn content in all film substrates remained virtually unchanged, since Zn^2+^ cations have a completed *d*-sublevel and are not sorbed by chitosan. The Mg concentration in our experimental chitosan films decreased during fungal cultivation. This is somewhat unexpected, since this element belongs to the alkaline earth metals of the second group and, like Zn, is sorbed by chitosan very poorly, or is not sorbed at all, as a result of which it was worth expecting the Mg content in the films remained unchanged after the growth of the fungus. It is quite possible that *F. oxysporum* could use not only magnesium from the culture medium during growth, but also Mg contained in the chitosan films. Since Mg^2+^ in the culture medium is more accessible for mycelial growth, it is also possible that this element may be washed out of the polymer matrix during the film sample transformation.

The absence of sorption capacity in the experimental films for the Fe^2+^ ions contained in the cultivation medium is logically explained by the loss of *N*-containing groups, including NH_2_– ones, and oxidation of the OH–groups involved in the chelation of chitosan complexes with metals, as well as by the crosslinking of macrochains and, accordingly, the loss of functional fragments coordinating chelate structures, as revealed by the FTIR spectroscopy data (Figure 5 and Figure S1) and organoelement analysis (Table 3). The influence of the increased degree of crystallinity of our experimental samples (Table 2 f) cannot be ruled out, since only the amorphous phase of the polymer participates in sorption. The decrease in free –OH and –NH_2_ groups in the CS-${\bar{M}}_{\eta }$·*FO* samples, which are adsorption centers for metal ion chelation, is confirmed by the increase in the time required to establish sorption equilibrium (from 1 h for CS-${\bar{M}}_{\eta }$ up to 24 h for CS-${\bar{M}}_{\eta }$·*FO*) and a significant decrease in the sorption capacity (by 60–85% depending on the samples) of Cu^2+^ ions, the adsorption of which by chitosan-containing sorbents in a neutral medium is extremely high (found in special experiments) [46].

### 3.4. Production of Extracellular Enzymes and Emulsifying Substances During Chitosan Film Transformation by F. oxysporum

**Figure 7:**
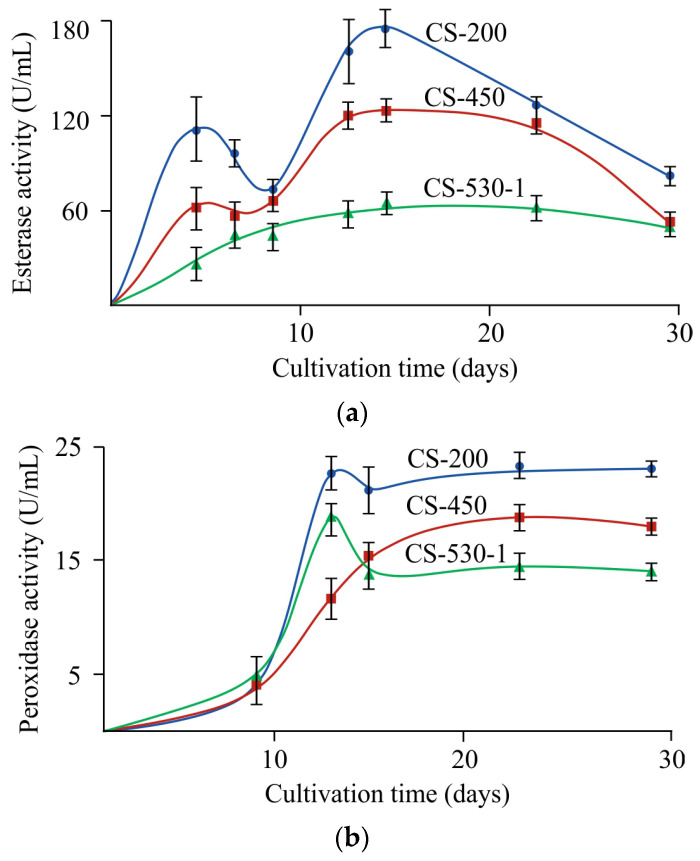
Hydrolase (**a**) and peroxidase (**b**) production during *F. oxysporum* growth on the CS-200, CS-450 and CS-530-1 films at 25 °C; the confidence interval of the mean values did not exceed 10%.

Chitosanases are the characteristic enzymes catalyzing chitosan depolymerization, and they predominantly cleave deacetylated sections of the aminopolysaccharide chain [4] to form chitooligomers [14]. In our study, no chitosanase activity was detected throughout the entire experiment and regardless of the conditions used. However, it was previously shown that *F. oxysporum* IBPPM 543 depolymerized polyethylene terephthalate using another hydrolytic enzyme (esterase). It was found that fungal growth on chitosan films was also accompanied by esterase production, starting from the 2^nd^–3^rd^ day of cultivation, reached a maximum on the 15^th^ day, and then decreased toward the end of the experiment, regardless of the molecular weight of chitosan in the films (Figure 7a).

In addition, natural polymer degradation by ascomycetes is often associated with the production of extracellular oxidases (laccases) and peroxidases (lignin peroxidase, Mn-peroxidase). In our experiments, no extracellular laccase activity was detected, and peroxidase production for the chitosan films of all ${\bar{M}}_{\eta }$’s was detected since day ~10 (later than for esterase), increased during the 13^th^ day of the experiment, and then reached a plateau (Figure 7b).

Using CS-530 as an example, the effect of film thickness and cultivation temperature on the activities of extracellular esterase and peroxidase at the end of the experiment was studied. It was found that the use of 100 μm thick film substrates led to an increase in esterase activity by 10% at 25 °C and had virtually no effect on this quantity at 30 °C. In the case of peroxidase, a two-fold decrease in the thickness of the initial film increased the enzyme activity at both cultivation temperatures: by 17 and 30% at 25 and 30 °C, respectively. If we compare the effect of temperature on the production of extracellular enzymes during fungal cultivation on film substrates of various thicknesses, it should be noted that their activity decreased with increasing temperature: for esterase by 25 and 32% and for peroxidase by 30 and 17% for the 200 μm and 100 μm thick films, respectively.

**Figure 8:**
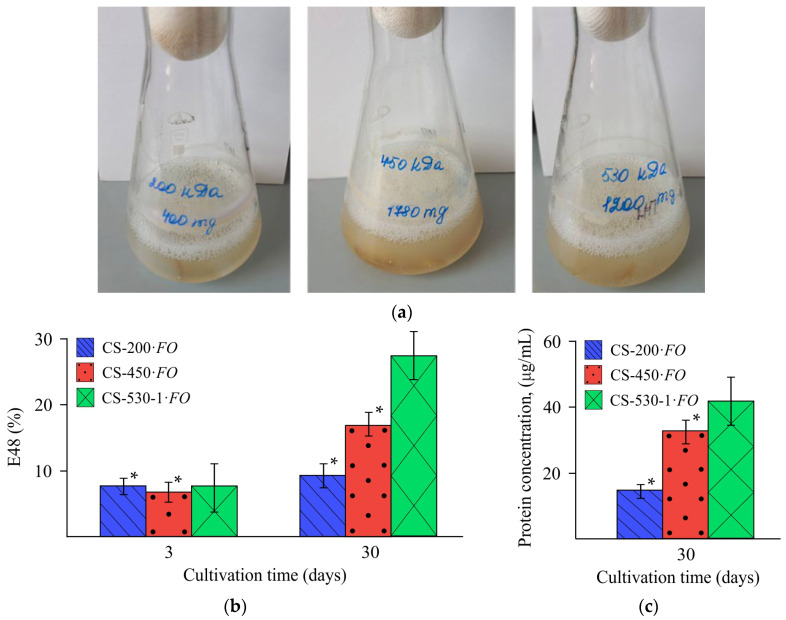
Characteristics of the culture medium during *F. oxysporum* growth in the presence of the CS-200, CS-450, and CS-530-1 films: photos of the corresponding objects (**a**), emulsifying activity on day 3 and 30 of the experiment (**b**), and extracellular protein concentration on day 30 of the experiment (**c**), at 25 °C. (*) means a statistically significant difference with the same indicator in the control experiment (*p* ≤ 0.05).

During fungal growth on chitosan films, foam formation was observed, which was a result of the production of emulsifying substances (Figure 8a). Estimating the emulsifying activity of the cultivation medium is one of the first stages of assessing biosurfactant production. In our experiments, the emulsifying activity (*E*_48_) increased significantly with the molecular weight of chitosan in the films utilized by the fungus (Figure 8b). The E_48_ value at the end of the experiment was 9.2, 17.0, and 27.4% for the CS-200·*FO*, CS-450·*FO,* and CS-530-1·*FO* films, respectively.

We also found an increase in the concentration of extracellular protein with an increase in the molecular weight of chitosan in the film substrate, namely, 15.0, 33.6, and 42.1 μg/mL for samples CS-200·*FO*, CS-450·*FO,* and CS-530-1·*FO*, respectively (Figure 8c).

### 3.5. Detection of the Activity of the Crude Enzyme Preparation Towards Chitosan Films

To study the catalytic effect of the detected extracellular enzymes on the structure and supramolecular ordering of chitosan in films, we used the CS-200 film and a crude enzyme preparation (the culture liquid containing esterase and peroxidase). The polymeric substrate was placed into the crude enzyme preparation without adding H_2_O_2_, which excluded the action of peroxidase, while esterase remained catalytically active. In the treatment of this experiment, catalytic amounts of H_2_O_2_ were added, and both enzymes retained their activity. At the end of the experiment, the film was washed with distilled H_2_O and dried to an air-dried state, and their structure and supramolecular ordering were analyzed using SEM, X-ray diffractometry, and FTIR spectroscopy.

After incubation of the CS-200 film with the crude enzyme preparation, as well as after *F. oxysporum* growth, a noticeable change in the morphology of the sample surface was observed (Figure 2g). Grain-like supramolecular formations of ovoid shape with characteristic effective diameters of ~1–2 μm appeared, between which pore-like defects with diameters of ~200–800 nm were observed. Numerous fairly large pores of spherical and oval shapes with the largest transverse size of ~1–6 μm were also recorded. The X-ray diffraction pattern of the CS-200·*FO^En^* film, like that of CS-200·*FO*, showed a significant enhancement of the equatorial reflections at 2Θ~10.4, 15.2, and 19.9 deg, the appearance of a new reflection at 2Θ~22.1 deg, and a significant increase in the degree of crystallinity of the polymer substrate (Figure 4a, CS-200·*FO^En^*; Table 2 f; and Table S1). A shift in the characteristic frequencies ν_as_ (CH), ν_s_ (CH), δ_as_ (CH), ν_C=O_ (Amide I), and ν_C–N_ (Amide III), the appearance of the bands δ_N–H_ (Amide II) and ν_C=O_ (CHO, COOH), and the presence of the band δ (C-1–H) characteristic of the initial CS-200 film were recorded in the FTIR spectrum of CS-200·*FO^En^*, as well as CS-200·*FO* (Figure 5, CS-200·*FO^En^*; Table S2). The results obtained indicate a uniform nature of the physicochemical structure formation in chitosan films after *F. oxysporum* growth during 30 days and after incubation with the crude enzyme preparation from *F. oxysporum* during 4 days.

## 4. Discussion

As is known, the presence of several types of functional groups in the chitosan macromolecule and its high sorption and complexing capacity contribute to modification of the structure and properties of the aminopolysaccharide and, accordingly, to the expansion of the areas of its practical application [1,2]. The highest reactivity is exhibited by the primary hydroxyl group (C6–OH) and amino group (C2–NH_2_) of the elementary unit, used for the synthesis of various *N*- and *O*-derivatives of chitosan [3,47,48]. The functional fragment C2–NH_2_ is effective in the processes of covalent crosslinking of macrochains when obtaining crosslinked structures with densely packed chains [49,50,51]. The –OH group at C3 is active in periodate or photocatalytic oxidation reactions, accompanied by cleavage of the bond between the C2 and C3 atoms of the glucopyranose ring and the formation of aldehyde and/or carbonyl groups [42,52]. The C6–OH fragments of macrochains also participate in photocatalysis or specific oxidation, oxidizing mainly to C=O without cleavage of the pyranose cycle [42,43]. Such oxidative processes are accompanied by a change in the C/H mass ratio and the degree of crystallinity of the sample, as well as an increase in the absorption band of the stretching vibrations of oxo groups (C=O) in the FTIR spectra. The glycosidic bond is destroyed by chemical or enzymatic hydrolysis [4,13,14,15]. The supramolecular ordering of chitosan is formed by several polymorphic modifications with a developed system of intra- and intermolecular contacts and a low degree of crystallinity, usually not exceeding 35–40% [41,53]. This aminopolysaccharide is one of the most effective natural polymeric ligands capable of binding a wide range of metal ions, with the exception of alkaline and alkaline earth metals having no free d- and f-orbitals [44,45]. Chitosan adsorption of transition metal ions, such as Cu(II) or Fe(III), occurs via a chelation mechanism involving the nitrogen atoms of amino groups and the oxygen atoms of hydroxyl groups [46]. In this case, the nitrogen atoms of the –NH_2_ groups are the main adsorption centers, while the –OH groups participate in coordination. The sorption capacity of chitosan with respect to metals is significantly affected by the degree of its crystallinity and the crosslinking of its molecular chains, which is due to a decrease in the number of free functional groups capable of participating in the sorption of metal ions [54].

Our experiments demonstrated the ability of the ascomycete *F. oxysporum* IBPPM 543 to use chitosan films as the sole source of carbon and energy, which is confirmed by the increase in the optical density of the cultivation medium and the active fouling of the polymer substrate. SEM recorded multiple defects in the morphostructure of the samples, showing the fungal mycelium growth not only on the surface but also in the bulk of the films. It was found that, after *F. oxysporum* growth, the films became more resistant to mechanical dispersion, but less durable and elastic under uniaxial tension. At the same time, the degree of crystallinity of chitosan significantly increased and its solubility in classical dissolving media (acetate buffer) decreased. In the process of overgrowing the films with the fungus, not only the supramolecular but also chemical structure of the aminopolysaccharide changed. FTIR spectroscopy showed chain crosslinking, modification, and even a loss of nitrogen-containing groups, as well as the occurrence of oxidative processes with the possible rupture of the glucopyranose ring, but without rupture of the *β*–1,4–glucosidic bond.

The nature of the physicochemical structure formation of polymer substrates is consistent with the elemental analysis of chitosan transformed by the fungus and the absence of its sorption capacity for metal ions, and is also confirmed by the enzymatic activities of the extracellular esterase and peroxidase detected, which are presumably involved in the crosslinking of macrochains and oxidative processes.

As already mentioned, we have detected no activity of chitosanase, the enzyme most often found in chitosan-degrading fungi. At the same time, we revealed an enzyme which actively hydrolyzed *p*-nitrophenyl butyrate, a typical substrate of fungal esterases, which belong to the α/β-hydrolase family and are capable of hydrolyzing high-molecular-weight synthetic polyesters, as well as low-molecular-weight soluble esters and short- and long-chain triacylglycerols [55]. These data suggest that esterase may be involved in the transformation of chitosan films by *F. oxysporum*.

The second enzyme identified, peroxidase, was produced at later stages of fungal growth. Similar dynamics of extracellular peroxidase production was noted during degradation of the synthetic polymer polyethylene terephthalate by the same strain of *F. oxysporum* [27]. Apparently, as in the case of polyethylene terephthalate, extracellular peroxidase is involved in the oxidation of metabolites or functional groups formed under the action of esterase. Note also that the highest activity of both extracellular enzymes was detected during cultivation of the fungus on the CS-200 film.

In addition to extracellular enzymes, the utilization of the film substrate was accompanied by the formation of foam, presumably due to the presence of a biosurfactant. Biosurfactant production by ascomycetes, including representatives of the genus *Fusarium*, is well known [56]. The biosurfactants produced by ascomycetes have different structures, namely, lipopeptides [57], glycolipids [58], enamine [56], and hydrophobin proteins [59]. The latter could facilitate attachment of the fungal mycelium to the hydrophobic surfaces of natural and synthetic polymers, thereby increasing their bioavailability, as shown in the process of polyethylene terephthalate degradation [60]. In our experiments, an increase in the concentration of extracellular protein with an increase in the ${\bar{M}}_{\eta }$ of the polymer and correlating with an increase in E_48_ may be an indirect confirmation of the protein nature of the revealed biosurfactant. Another argument in favor of this assumption may be the fact that the fungi form a mycelial ring attached to the surface of the flask and overgrow the chitosan films. Apparently, the biosurfactants we revealed could also promote the accessibility of functional fragments of the macromolecular chains of the aminopolysaccharide for enzymes. This assumption will be the subject of further research.

In addition, the results obtained in the study of the processes occurring during the treatment of the film substrate with an enzyme preparation of esterase and peroxidase indicate a uniform nature of the physicochemical structure formation in chitosan films after the growth of *F. oxysporum* for 30 days and after incubation with the crude enzyme preparation from *F. oxysporum* for 4 days.

The data obtained indicate specificity of the aminopolysaccharide transformation processes in the *F. oxysporum* culture, which differ from the traditional processes of fungal chitosan depolymerization under the action of chitosanases. The quantitative indicators of micromycete growth and the activity of the enzymes produced are affected by the molecular weight of chitosan, the polymer film thickness, and the temperature of the cultivation medium.

## 5. Conclusions

The results obtained can be considered as a fundamental basis for the development of new classes of chitosan-containing biocomposites using mycotechnologies, for example, polymeric materials for medical and biological purposes to be stable in acidic buffer media, covering and packaging films for food products, frost-protective coatings for winter crop seeds, etc. The high resistance of films modified by *F. oxysporum* to dispersion seems very promising in the development of chitosan-containing tissue-engineered structures to ensure their mechanical support during tissue restoration. The production of specific extracellular enzymes which do not destroy chitosan produced by the fungus expands biotechnological approaches to polymer modification to form biomaterials with new functional properties. The uniform nature of the physicochemical modification of chitosan in the ascomycete *F. oxysporum* medium and the crude enzyme preparation based on hydrolase and peroxidase allow using these enzymes to modify the polymer without participation of the fungal culture.

## Figures and Tables

**Table 1:** Characteristics of the initial chitosan powders.

Chitosan Sample	Viscosity-Average Molecular Weight $\;{\bar{M}}_{\eta }$, kDa	Degree of Deacetylation, mol.%	Bulk Density, g/cm^3^	Moisture Content, wt.%	Ash Content, wt.%
CS-200	200	82.0	0.32	9.6 ± 0.5	0.58
CS-450	450	79.0	0.42	8.9 ± 0.2	0.53
CS-530	530	80.0	0.44	9.8 ± 0.4	0.53

**Table 2:** Physicochemical characteristics and strength parameters of our chitosan films: initial ones (I, CS-${\bar{M}}_{\eta }$), after *F. oxysporum* growth for 30 days at 25 and 30 °C (II and II*, respectively, CS-${\bar{M}}_{\eta }$·*FO*) and after incubation with the crude enzyme preparation from *F. oxysporum* at 25 °C for 4 days (III, CS-200-*FO^En^*).

Parameter		Film Type	Sample			
			CS-200	CS-450	CS-530-1	CS-530-2
Physicochemical parameters						
(a)	Thickness, μm	I	200 ± 15			100 ± 10
		II	300 ± 30	550 ± 30	750 ± 40	180 ± 25
(b)	Weight loss, %	II	7.1 ± 1.8	2.6 ± 0.4	3.9 ± 0.6	2.8 ± 0.6
(c)	Humidity, %	I	8.4 ± 2.2	8.7 ± 1.8	7.5 ± 2.2	7.9 ± 1.1
		II	7.7 ± 1.4	8.6 ± 3.6	7.3 ± 1.4	7.8 ± 1.3
(d)	Color	I	Light beige			
		II	Ochre brown			
(e)	Insoluble fraction, wt.%	I	No color			
		II	75 ± 5	82 ± 3	95 ± 2	87 ± 1
		II*	–	–	87 ± 3	82 ± 2
(f)	Crystallinity degree χ, %	I	32	38	38	40
		II	46	57	44	38
		II*	n.d.	n.d.	46	42
		III	48	n.d.	n.d.	n.d.
Strength parameters						
(g)	Tensile strength σ_p_, MPa	I	12.2 ± 3.0	15.6 ± 3.6	14.8 ± 2.7	n.d.
		II	5.3 ± 0.8	9.1 ± 0.6	10.0 ± 1.5	n.d.
(h)	Relative elongation at break ε_p_, %	I	90 ± 10	80 ± 15	65 ± 15	n.d.
		II	55 ± 5	60 ± 3	40 ± 5	n.d.
(i)	Young’s modulus E, MPa	I	11.9 ± 2.1	4.3 ± 1.0	4.1 ± 1.9	n.d.
		II	4.2 ± 1.4	3.9 ± 1.4	3.8 ± 0.9	n.d.

n.d.—not determined.

**Table 3:** Elemental analysis data for our chitosan films: the initial ones (I, CS-${\bar{M}}_{\eta }$) and those after *F. oxysporum* growth at 25 °C for 30 days (II, CS-${\bar{M}}_{\eta }$·*FO*).

Sample	Film Type	Element Content, wt.%			C/N	C/H
		C	H	N		
CS-200	I	39.94	6.36	6.31	6.3	6.3
	II	37.20	6.01	3.30	11.3	5.7
CS-450	I	38.70	6.83	5.73	6.8	5.7
	II	36.20	6.47	3.07	11.8	5.6

## Data Availability

The raw/processed data can be provided when required.

## Supplementary Materials

The following supporting information can be downloaded at: https://www.mdpi.com/article/10.3390/jof11080565/s1, Table S1: Diffraction reflection angles and interplanar distances in crystallites of our chitosan films: initial (I) and after *F. oxysporum* growth for 30 days at 25 (II) and 30 °C (II*) and after incubation with the crude enzyme preparation from *F. oxysporum* (CS-${\bar{M}}_{\eta }$·*FO^En^*) for 4 days at 25 °C (III); Table S2: Correlation of absorption bands in the FTIR spectra of dispersed samples of the initial CS-200 film (I), CS-200·*FO* film (II), and its insoluble fraction CS-200·*FO^InsF^* (III) after *F. oxysporum* growth for 30 days at 25 °C and of CS-200·*FO^En^* film after incubation with the crude enzyme preparation from *F. oxysporum* for 4 days at 25 °C (IV); Figure S1: FTIR spectra of dispersed CS-450 (a) and CS-530-1 (b) films: initial CS-${\bar{M}}_{\eta }$ film, CS-${\bar{M}}_{\eta }$·*FO* film, and the insoluble fraction CS-${\bar{M}}_{\eta }$·*FO^InsF^* after *F. oxysporum* growth for 30 days at 25 °C.

## Abbreviations

The following abbreviations are used in this manuscript:

CS	chitosan
*En*	enzyme
*FO*	*F. oxysporum*
*InsF*	insoluble fraction
IR	infrared spectroscopy
FTIR	Fourier-transform infrared spectroscopy
SEM	scanning electron microscopy
