# Supplementary material for: Esterase and Peroxidase Are Involved in the Transformation of Chitosan Films by the Fungus Fusarium oxysporum Schltdl. IBPPM 543
Source: J Fungi (Basel). 2025 Jul 29;11(8):565. doi: 10.3390/jof11080565 (PMC12387507; doi:10.3390/jof11080565)
Supplement: Supplementary file 1 [file jof-11-00565-s001.zip › jof-3734979-supplementary.pdf]

Supplementary materials

Transformation of chitosan films by the fungus *Fusarium oxysporum* Schltdl.

Natalia N. Pozdnyakova[1,2*], Tatiana S. Babicheva[1], Daria S. Chernova[1], Irina Yu. Sungurtseva[1,2], Andrei M. Zakharevich[1], Sergei L. Shmakov[1], and Anna B. Shipovskaya[1]

[1] *Institute of Chemistry, Saratov National Research State University named after N.G. Chernyshevsky, Astrakhanskaya 83, Saratov 410012, Russian Federation,* shipovskayaab@yandex.ru

[2] *Institute of Biochemistry and Physiology of Plants and Microorganisms, Saratov Scientific Centre of the Russian Academy of Sciences (IBPPM RAS), prosp. Entuziastov 13, Saratov 410049, Russian Federation,* pozdnyakova_n@ibppm.ru

Designation and characteristics of the source chitosan powders

Chitosan sample	Viscosity-average molecular weight $\bar{M}_\eta$, kDa	Degree of deacetylation, mol.%
CS-200	200	82.0
CS-450	450	79.0
CS-530	530	80.0

Table S1. Diffraction reflection angles and interplanar distances in crystallites of our chitosan films: initial (I) and after *F. oxysporum* growth for 30 days at 25 (II) and 30°C (II*), after incubation with a crude enzyme preparation of *F. oxysporum* (CS-$\overline{M}_\eta \cdot FO^{En}$) for 4 days at 25°C (III).

Film type	Sample							
	CS-200		CS-450		CS-530-1		CS-530-2	
	Parameters							
	Θ, deg	*d*, Å	Θ, deg	*d*, Å	Θ, deg	*d*, Å	Θ, deg	*d*, Å
I	10.1	8.8	10.5	8.4	10.1	8.8	10.4	8.5
	15.1	5.9	–	–	15.7	5.6	15.0	5.9
	19.7	4.5	20.5	4.3	19.7	4.5	19.8	4.5
II	10.3	8.7	9.9	8.9	10.1	8.8	9.5	9.3
	14.9	6.0	–	–	–	–	–	–
	19.8	4.5	19.7	4.5	19.8	4.5	20.4	4.3
	22.1	4.1	21.8	4.1	22.1	4.0	–	–
II*	n.d.				10.5	8.4	10.3	8.6
					20.2	4.4	20.2	4.4
					22.3	4.0	22.4	3.9
III	10.4	8.5	n.d.					
	15.2	5.8						
	19.9	4.5						
	22.1	4.1						

n.d. – not determined

Table S2. Correlation of absorption bands in the FTIR spectra of dispersed samples of the initial CS-200 film (I), CS-200·*FO* film (II) and its insoluble fraction CS-200·*FO*^{InsF} (III) after *F. oxysporum* growth for 30 days at 25°C, CS-200·*FO*^{En} film after incubation with a crude enzyme preparation of *F. oxysporum* for 4 days at 25°C (IV).

	Vibration type of the structural fragment	Film type CS-200			
		I	II	III	IV
		Absorption band frequency, cm^{-1}			
1	$\nu_{O–H}$, $\nu_{N–H}$	3700–3300			
2	ν_{as} (CH)	2922	2924	2925	2925
3	ν_{s} (CH)	2857	2855	2853	2854
4	$\nu_{C=O}$ (CHO, COOH)	–	1736		
5	$\nu_{C=O}$ (Amide I)	1630	1633	1630, 1609	1642
6	$\delta_{N–H}$ (Amide II)	–	–	1560	1562
7	$\nu_{C–O}$ (COO^{-})	1460			
8	δ_{sc} (CH)	1422	1420	1400, 1385	1422
9	δ_{as} (CH)	1381	1383	1385	1375
10	$\nu_{C–N}$ (Amide III)	1323	1320	1313	1316
11	ν_{CO}, δ_{CO} (OH)	1262	1262	1261	1256
12	Glucopyranose ring: ν (C–C), ν (C–O), ν (C–N)	1170–840			
	Glucopyranose ring: δ (C-1–H)	896			876

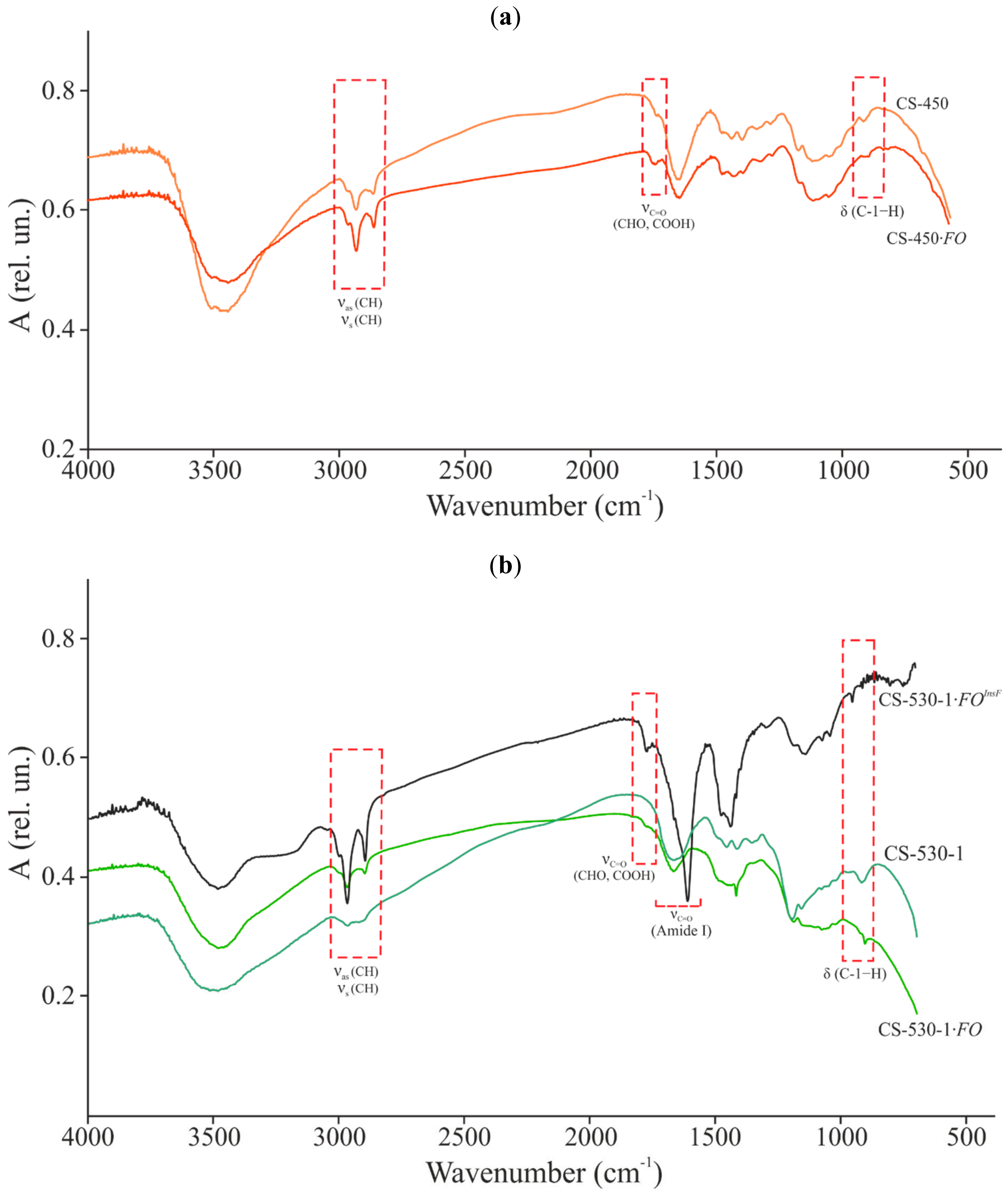

Figure S1. FTIR spectra of dispersed CS-450 (**a**) and CS-530-1 (**b**) films: initial CS-$\bar{M}_\eta$ film, CS-$\bar{M}_\eta \cdot FO$ film and the insoluble fraction CS-$\bar{M}_\eta \cdot FO^{InsF}$ after *F. oxysporum* growth for 30 days at 25°C.
